# Understanding the nervous system: lessons from Frontiers in Neurophotonics

**DOI:** 10.1117/1.NPh.11.1.014415

**Published:** 2024-03-27

**Authors:** Yves De Koninck, Johanna Alonso, Stéphane Bancelin, Jean-Claude Béïque, Erik Bélanger, Catherine Bouchard, Marco Canossa, Johan Chaniot, Daniel Choquet, Marie-Ève Crochetière, Nanke Cui, Lydia Danglot, Paul De Koninck, Anna Devor, Mathieu Ducros, Angela M. Getz, Mohamed Haouat, Iván Coto Hernández, Nate Jowett, Iason Keramidis, Céline Larivière-Loiselle, Flavie Lavoie-Cardinal, Harold D. MacGillavry, Asiye Malkoç, Mattia Mancinelli, Pierre Marquet, Steven Minderler, Maxime Moreaud, U. Valentin Nägerl, Katerina Papanikolopoulou, Marie-Eve Paquet, Lorenzo Pavesi, David Perrais, Romain Sansonetti, Martin Thunemann, Beatrice Vignoli, Jenny Yau, Clara Zaccaria

**Affiliations:** a CERVO Brain Research Centre, Québec City, Québec, Canada; b Laval University, Department of Psychiatry and Neurosciences, Faculty of Medicine, Québec City, Québec, Canada; c University of Bordeaux, Interdisciplinary Institute for Neuroscience, National Centre for Scientific Research (CNRS), Bordeaux, France; d University of Ottawa, Brain and Mind Research Institute, Centre of Neural Dynamics, Ottawa, Ontario, Canada; e Laval University, Département de physique, de génie physique et d’optique, Québec City, Québec, Canada; f Laval University, Institute Intelligence and Data, Québec City, Québec, Canada; g University of Trento, Department of Cellular Computational and Integrative Biology, Trento, Italy; h University of Bordeaux, CNRS, Institut national de la santé et de la recherche médicale (INSERM), Bordeaux Imaging Center (BIC), Bordeaux, France; i Harvard Medical School, Surgical Photonics & Engineering Laboratory, Mass Eye and Ear, Boston, Massachusetts, United States; j Université Paris Cité, Institute of Psychiatry and Neuroscience of Paris, INSERM U1266, Paris, France; k Laval University, Department of Biochemistry, Microbiology, and Bioinformatics, Faculty of Science and Engineering, Québec City, Québec, Canada; l Boston University, Department of Biomedical Engineering, Boston, Massachusetts, United States; m Massachusetts General Hospital, Athinoula A. Martinos Center for Biomedical Imaging, Charlestown, Massachusetts, United States; n Utrecht University, Faculty of Science, Division of Cell Biology, Neurobiology and Biophysics, Department of Biology, Utrecht, The Netherlands; o University of Trento, Department of Physics, Trento, Italy; p Laval University, Centre d’optique, photonique et laser (COPL), Québec City, Québec, Canada; q IFP Energies nouvelles, Solaize, France; r Institute for Fundamental Biomedical Research, Biomedical Sciences Research Center Alexander Fleming, Vari, Greece

**Keywords:** Frontiers in Neurophotonics, neurophotonic technology, synaptic function, optical instrumentation

## Abstract

The Frontiers in Neurophotonics Symposium is a biennial event that brings together neurobiologists and physicists/engineers who share interest in the development of leading-edge photonics-based approaches to understand and manipulate the nervous system, from its individual molecular components to complex networks in the intact brain. In this Community paper, we highlight several topics that have been featured at the symposium that took place in October 2022 in Québec City, Canada.

## 1 Introduction: The Frontiers in Neurophotonics Symposium and Summer School

Understanding the nervous system requires an integrated comprehension of its building blocks, including its cells, their structural and functional connections, their mode of operations at the molecular level, and how they are organized in networks to sense, decode, respond, and instruct/control the rest of the body. Advances in understanding cell function critically depend on our capacity to improve the resolution of dynamic molecular mechanisms and the availability of the appropriate tools to study these events in live cells and intact tissue. This is particularly true in the field of neurosciences, where challenges in spatial and temporal resolution are pushed to the extreme. These challenges include: breaking the diffraction limits of light to follow molecular events in submicron scale dendritic spines or to understand the mechanics of neurotransmitter release in synaptic terminals; following the spatiotemporal dynamics of signaling proteins on highly fluid membranes; developing novel probes to detect enzyme activity in real time *in situ*; recording simultaneously the activity of multiple nerve cells, at millisecond time scales, within a large area to decipher network interactions; probing deeper and deeper into the brain; monitoring intrinsic cellular events at high resolution with minimal invasiveness in intact animals; achieving label-free chemical imaging in live tissue; exploiting light to control neuronal activity in specific brain nuclei in freely moving animals; and more.

**Fig. 1:**
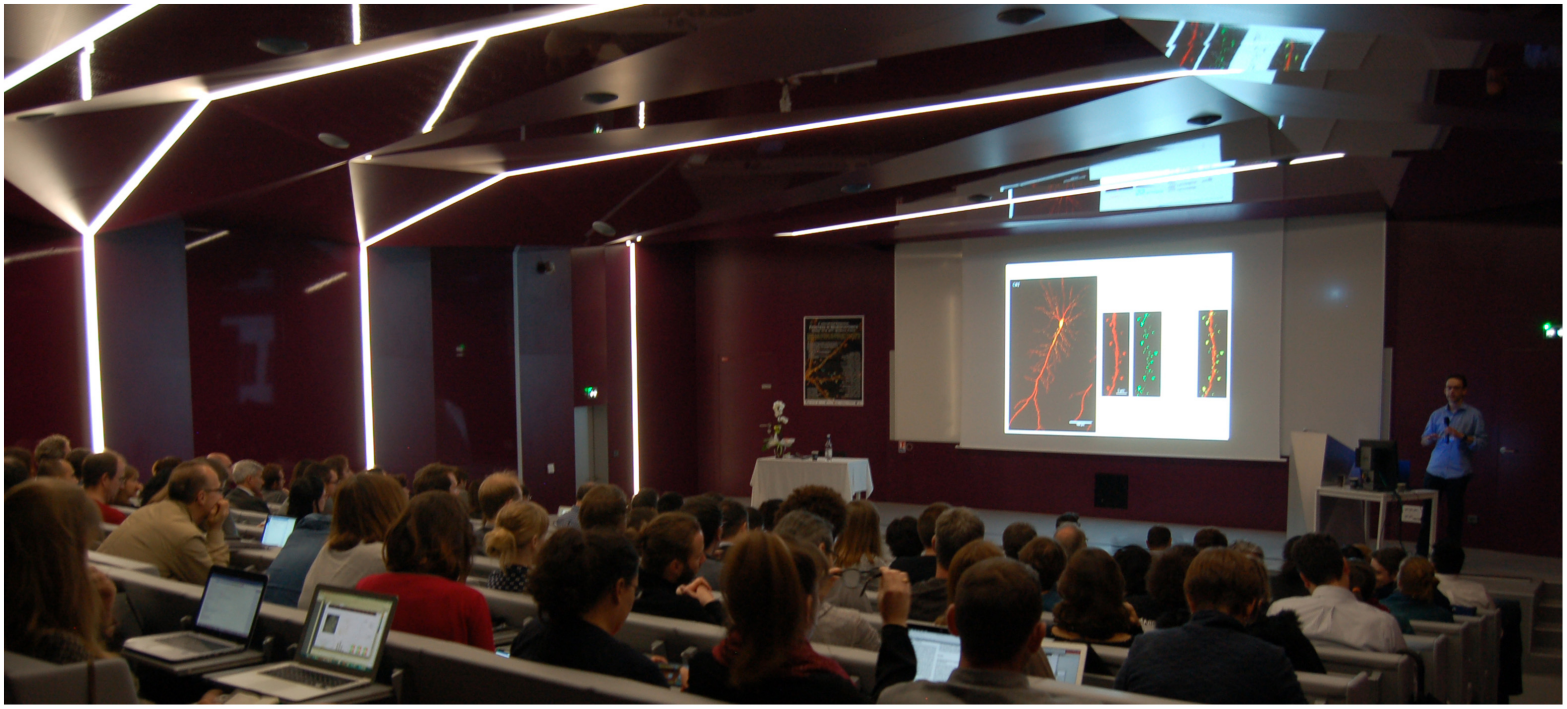
Frontiers in Neurophotonics Symposium 2022.

Starting in 2008, these transdisciplinary challenges and the proposed solutions have been presented and debated at the Frontiers in Neurophotonics Symposium jointly organized by Université Laval (Canada) and Université de Bordeaux (France) (Fig. 1). The meeting has been taking place approximately every two years, bringing together neurobiologists and physicists/engineers who share interest in the development of leading-edge photonics-based approaches to understanding and manipulating the nervous system, from its individual molecular components to complex networks in the intact brain. The meeting showcases new methodology demonstrating how novel neurophotonic methods are leading to fascinating discoveries and stimulating conceptual advances. The themes and methods covered during the meeting range from measuring single molecule dynamics at synapses to imaging sensory processing in intact brains, including:

• Nanophotonics probes for biosensing and molecular tracking
• Molecular dynamics in nanoscale compartments
• Monitoring molecular interactions in live neurons
• Non-linear optics for high resolution, *in vivo* deep-tissue imaging
• Overcoming temporal resolution challenges for optical monitoring of network activity
• Nanoscopy: super-resolution optical imaging at live synapses
• Intrinsic, label-free molecular imaging and spectroscopy
• Fiber probes for sensing, imaging, and stimulating
• Microendoscopy
• Optical tomography
• Photoablation techniques
• Multimodal imaging
• Optogenetics and photomanipulation
• Image analysis and computational approaches
• Application of photonics to neurosurgery

With this wide and immersive scope, the Frontiers in Neurophotonics Symposium has, in the last sixteen years, cultivated a multidisciplinary community, fostered new ideas and collaborations, and offered a forum for students and fellows to discuss their work with leading experts and peer trainees. In 2025, the 7^th^ edition of the symposium will take place at Université de Bordeaux.

**Fig. 2:**
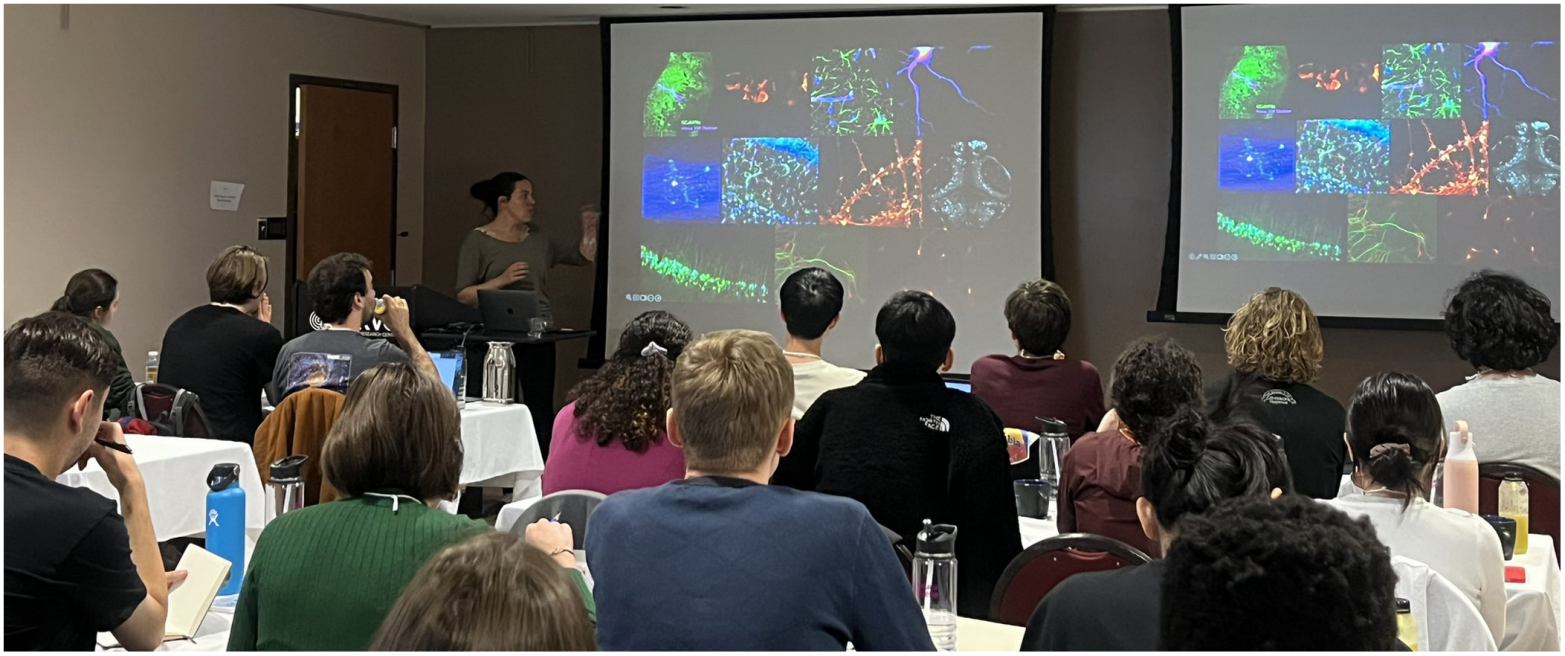
Flavie Lavoie-Cardinal introducing the principles of fluorescence at Frontiers in Neurophotonics International Summer School 2023.

Complementary to the symposium and drawing from the same community of experts, the annual Frontiers in Neurophotonics International Summer School has provided practical knowledge to doctoral and postdoctoral trainees around the world in data acquisition, data processing, appropriate interpretation, and understanding the underlying physical principles of operation (Fig. 2). The goal of the school has been to ensure that trainees become versed in the neurophotonic tools and leave the program with sufficient “know-how” expertise to successfully carry out neuroimaging projects in their home institutes and universities. In 2024, the 17^th^ edition of the school is taking place at the CERVO Brain Research Center in Québec City.

Given the rapid growth and evolution of neurophotonic technologies, a comprehensive account of the tools and applications discussed at the symposium and taught at the school over the years would be a massive undertaking. Instead, we choose to highlight several topics that have been featured at the last symposium that took place in October 2022. We hope that this sampler will make our readers interested in learning more and participating in these events!

## 2 Optical Contrasts and Probes

### 2.1 Label-Free Live Cell Imaging

**Fig. 3:**
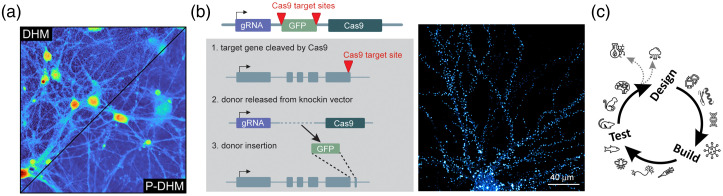
Intrinsic and engineered optical contrasts. (a) Label-free imaging: split-view of a neuronal cell culture comparing a classical DHM image (top left) with an image acquired using the automated P-DHM (bottom right). (b) CRISPR/Cas9-based tagging of endogenous proteins. Left: knock-in vector (top) and strategy (bottom). Right: synaptic protein Shank2 knock-in neuron. Adapted from Ref. 13. (c) Optimization of optical biosensors in a feedback loop between developers and testers.

Label-free imaging relies on interactions of light with brain tissue in the absence of any extrinsic (synthetic or genetically encoded) probes. Quantitative-phase imaging (QPI) is a label-free imaging modality that has been used for imaging of live cells.^1^ Nowadays, one of its most popular implementations is digital holographic microscopy (DHM) due to its computational flexibility originating from the numerical reconstruction of the wavefront up to the plane of the specimen.^2^ By fully simulating light-wave propagation and conditioning, it opens the possibility for autofocusing,^3^ thereby compensating thermal and experimental drifts,^4^ enabling total correction of aberrations and distortions,^5^ and allowing extended depth-of-focus.^6^ Due to its interferometric nature, the quantitative-phase signal (QPS) of DHM is extremely accurate, allowing sensitivity down to the nanometer scale.^7^ As a single shot technique, DHM is particularly well suited to capturing dynamic biological processes.^8^ Moreover, to preserve the size of the field-of-view,^9^ especially in an off-axis configuration and to record holograms exhibiting well-defined interference fringes, a prerequisite for accurate and precise phase retrieval,^10^^,^^11^ a light source with a certain level of coherence is necessary. However, the coherence property of the light source itself generates coherent noise (CN), an acknowledged source of image quality limitation which mitigates some of the many advantages of DHM.^12^ The CN causes a well-recognized granular appearance in quantitative-phase images, obscuring thin and small cellular structures. Polychromatic DHM (P-DHM) has the ability to drastically reduce CN in quantitative-phase images and is particularly effective at revealing fine cellular processes, such as neuronal connections and neurites [Fig. 3(a)].^14^ Ongoing work using deep learning is attempting to make P-DHM noise reduction accessible to conventional digital holographic microscopes.

### 2.2 Labeling of Endogenous Proteins with CRISPR/Cas9-Based Genome Editing

Mapping the distribution and dynamics of proteins is key for a mechanistic understanding of neuronal functioning.^15^ Astonishing progress has been made in fluorescence microscopy techniques that can now resolve subcellular protein organization at increasingly high resolution. Yet, the power of these imaging techniques ultimately relies on methods that accurately tag proteins without adverse side effects. Standard labeling approaches in the field often rely on overexpression of exogenous tagged proteins that can have detrimental effects on neuronal structure and function. An alternative approach that overcomes this limitation is to label endogenous proteins in neurons.^16^

This can be achieved using CRISPR/Cas9-based genome editing to modify, remove or add DNA segments to the genome. With the CRISPR/Cas9 vector system, one can insert epitope tags in specific genes and generate a library of CRISPR/Cas9 knock-in vectors to tag a range of synaptic, trafficking, and signaling proteins in neurons [Fig. 3(b)].^13^ This allows tagging of proteins for standard microscopy applications, as well as super-resolution microscopy, in cultured neurons and *in vivo.* It can then be used to study cell-type- and synapse-type-specific regulatory mechanisms controlling protein distribution and function. To analyze the spatial distribution of proteins both in conventional and super-resolution imaging one can use SODA (Standard Object Distance Analysis) plugin — a free software able to detect colocalization and apposition.^17^ In the context of synapses, SODA enables the detection of pre- and post-synaptic protein alignment.

### 2.3 Dissemination of Novel Optical Biosensors

For true impact, new tools should not be restricted to few highly specialized labs with niche expertise but serve the collective imagination of the largest collection of labs possible. One can facilitate adoption of new technologies by non-expert labs by building an active feedback loop between the developing and testing teams. In this context, the Canadian Optogenetics and Vectorology Foundry (neurophotonics.ca/COVF) has developed a BioFoundry based on a design-build-test (DBT) cycle where groups dedicated to design and development collaborate with series of testers across biological model systems [Fig. 3(c)].^18^ Tools developed by this BioFoundry include the near infrared calcium sensor NIR-GECO^19^^,^^20^ and the lactate sensor LACCO.^21^

## 3 Imaging and Manipulating Synaptic Function

The number and nanoscale organization of neurotransmitter receptors localized at the post-synaptic density are key determinants of the efficacy of synaptic transmission, while the rapid and dynamic spatial regulation the receptors constitute a major mechanism underlying the plasticity of synaptic transmission.^22^ The complex subcellular morphology and connectivity patterns of neurons, combined with the optical scattering properties of brain tissue and high signal density of protein(s) of interest, create technical limitations for studying the mechanisms of synaptic function in live tissue preparations. However, recent developments in advanced imaging methods and molecular approaches to label or functionalize proteins of interest have brought these questions within reach of experimental neuroscientists.^15^ Among these advances are lattice light sheet microscopy (LLSM) combined with CRISPR-Cas9-assisted fluorescent tagging of endogenous proteins, fluorescence microscopy of single exocytosis and endocytosis events in neuronal dendrites exploiting the tagging of post-synaptic receptors with pH-sensitive fluorescent proteins, and continuing development and optimization of optogenetic tools.

### 3.1 Lattice Light Sheet Microscopy

The study of dynamic neurobiological processes, such as synaptic plasticity, requires imaging of live samples at the sub-cellular level with high temporal resolution and minimal phototoxicity. LLSM^23^ specifically addresses these needs. LLSM has been used to image neuronal activity in brain slices.^23^^–^^26^ However, imaging at high resolution and contrast inside an opaque bio-tissue, such as brain slice, is a challenging task. Indeed, both the excitation and detection optical paths are subject to aberrations because of inhomogeneities of refraction indices. In particular, the light sheet is deflected, such as a blurred image of the sample is projected onto the camera. These deleterious effects can be mitigated using active image improvement techniques that have been developed for light sheet microscopes including auto-focusing and wavefront correction with adaptive optics.^27^^–^^29^

**Fig. 4:**
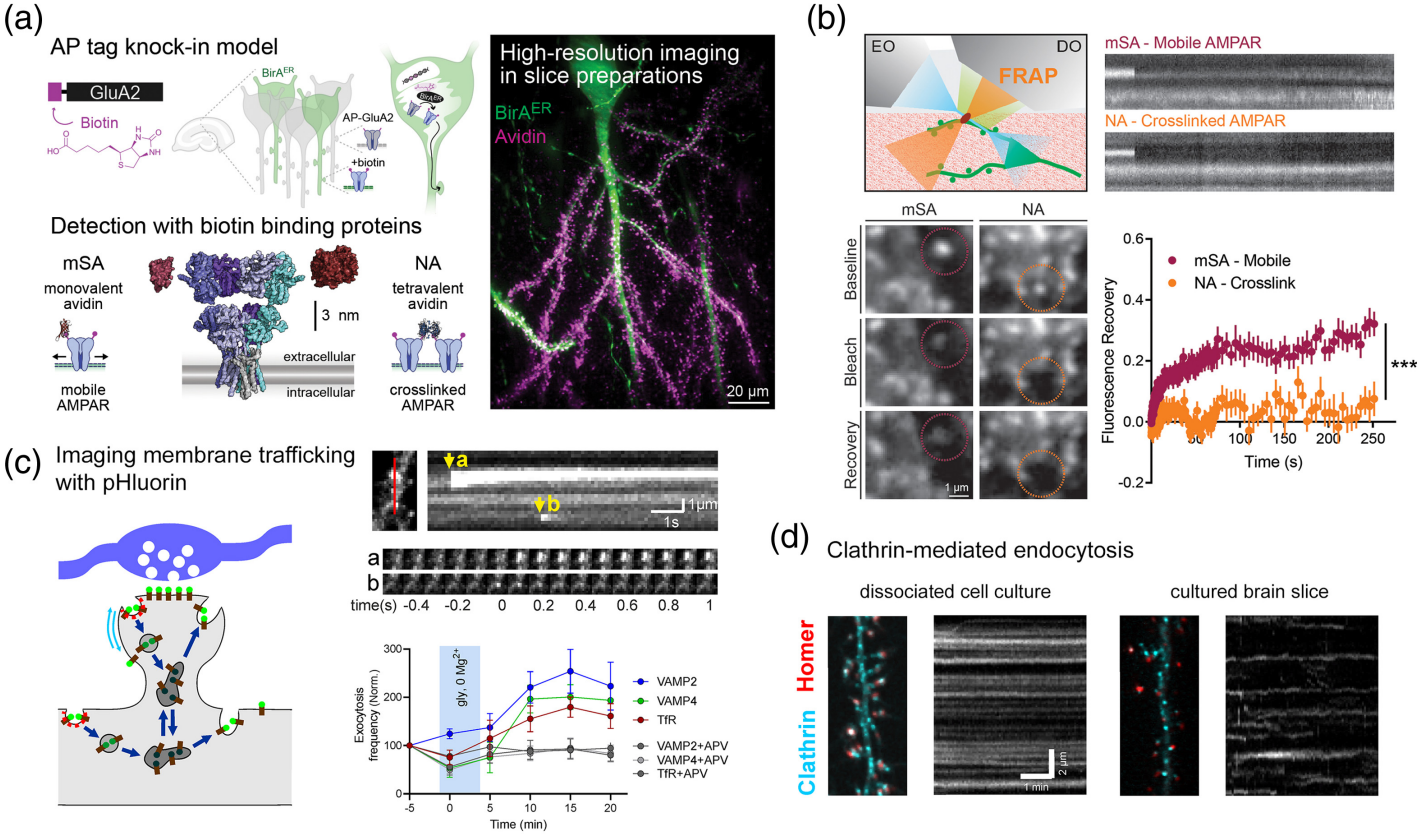
Live imaging of synaptic function. (a), (b) FRAP imaging of AMPAR diffusion. (a) Schematics of GluA2 labeling (left) and a reconstructed LLSM image of a GFP-expressing CA1 pyramidal neuron with labeled GluA2. (b) FRAP experiments performed with PSM. Top left: FRAP schematics. Top right: fluorescence recovery kymographs. Bottom left: spine regions (dashed circles) before (baseline) and after targeted photobleaching (bleach) and diffusion-dependent recovery (recovery). Bottom right: fluorescence recovery time-courses. Adapted from Ref. 24. (c), (d) Imaging of membrane trafficking with pH-sensitive pHluorin. (c) Left: schematics showing SEP-labeled cargo (brown sticks), visible at neutral pH (green lollipops) but not at the acidic pH of REs (dark gray). Blue, presynaptic terminal; gray, post-synaptic spine. Top right: kymograph showing detection of exocytosis events (top). Bottom right: an increase in exocytosis following long term potentiation (LTP). (d) Images neuronal dendrites transfected with Homer1c-tdTomato and clathrin-GFP and kymographs of clathrin-GFP showing that clathrin-coated structures are stable in cultured neurons but transient in the slice. Adapted from Ref. 34.

Combined with CRISPR-Cas9 genome editing, LLSM allows imaging of endogenous proteins, such as the AMPA subtype of glutamate receptors (AMPAR). One can measure the surface diffusion of endogenous AMPARs using a knock-in mouse model with a functionalized GluA2 subunit that allows sparse labeling of AMPARs in slice preparations and *in vivo*.^24^ In this mouse model, CRISPR-Cas9 genome editing was used to knock-in the 15 amino acid biotin acceptor peptide tag (AP) on the extracellular N-terminus of GluA2 (AP-GluA2). Labelling specificity of AP-GluA2 containing AMPAR was achieved by target-specific expression of biotin ligase (BirA), which selectively biotinylates the AP tag.^30^^,^^31^ This in turn allows for the use of small, high-affinity biotin binding proteins (avidins) conjugated to fluorescent dyes to monitor the surface mobility of endogenous AMPAR. Due to their small size (∼3-6  nm), avidins efficiently target membrane proteins in organized brain tissue and confined synaptic domains.^32^^,^^33^ Because the AP-tag knock-in strategy can be broadly adapted for the study of cell surface proteins, the same labeling approach can be applied to a wide range of biological research questions. Further, fluorescent labeling of endogenous synaptic proteins, such as AMPAR, in LLSM can be combined with a photo-stimulation module (PSM) for all-optical synaptic physiology experiments,^23^^,^^24^ including fluorescence recovery after photobleaching (FRAP) imaging of AMPAR diffusion dynamics^24^ [Figs. 4(a)-4(b)]. These new molecular tools and high-resolution imaging techniques enable detailed studies of synaptic organization and plasticity.^15^^,^^22^

### 3.2 Imaging Endo- and Exocytosis of Proteins Tagged with pH-Sensitive Fluorescent Labels

Membrane trafficking of post-synaptic receptors is a key determinant of synaptic transmission and synaptic plasticity. Imaging individual trafficking steps in the post-synaptic neuron is thus important to perform for understanding the cellular mechanisms of synapse regulation. Specifically, the imaging of single exocytosis and endocytosis events in neuronal dendrites is best achieved by tagging transmembrane proteins, such as post-synaptic receptors, with pH-sensitive fluorescent proteins, including the superecliptic pHluorin (SEP), in their extracellular portion.^34^ Exocytosis is detected by the transition in pH, from acidic (5.5, inside an acidic vesicle) to neutral (7.4, extracellular medium). Therefore, one can map exocytic events relative to spines and PSDs and assess the modulation of exocytosis events during the induction of synaptic plasticity. For example, the direct regulation of exocytosis mode by L-type calcium channels^35^ or the contribution of various classes of recycling endosomes^36^ were shown to be involved in long-term potentiation (LTP).

Detection of endocytosis with the highest spatial and temporal resolution is achieved by testing with repetitive pH changes (or pulsed pH, ppH) the accessibility of newly formed endocytic vesicles containing SEP-tagged receptors. It is then possible to map these endocytic events relative to PSDs and show the transient increase in endocytosis frequency following the induction of LTD.^37^^,^^38^ These imaging modalities have been so far used mostly in cultured neurons. It is now possible to use sensitive imaging modalities, such as LLSM, to image endocytic structures in living tissue^28^^,^^34^ [Figs. 4(c)–4(d)]. Together with genome editing strategies to tag with SEP endogenous receptors or transporters,^16^^,^^39^^–^^41^ these new approaches are paving the way to a detailed understanding of the trafficking steps at play in intact neuronal networks.

### 3.3 Optogenetics and Synaptic Function

Optogenetic techniques take advantage of the exquisite levels of cellular control that are enabled using the combination of light and genetically targeted constructs. They are becoming increasingly mainstream in neuroscience laboratories and there is hope for therapeutic applications in the future. However, simultaneous stimulation of more than a single location (a neuron or a synapse) is usually implemented using complex optical setups. On the other hand, for cell culture applications, one can reduce the complexity using a digital light processor (DLP) with an integrated digital micromirror device (DMD) aligned to the EPI-fluorescence port of a spinning disk microscope. Thanks to the DMD, the DLP device can create custom spatial light patterns with a resolution down to ∼3  μm with a 20x objective.^42^ This platform can be coupled with optical or electrophysiological monitoring to perform experiments involving the selective excitation/inhibition of a few cells in a neuronal network, followed by the molecular, physiological, and morphological characterization. Reductionistic by design, the platform can be used to investigate basic processes occurring in neuronal cultures at the cellular and synaptic levels, otherwise difficult to address *in vivo*.

As in the case of fluorescent biosensors, addressing specific biological questions often requires application-specific optimization, which often exceeds the expertise and capabilities of one team. Therefore, an easy adoption can be facilitated by push-pull collaboration between a series of Cores and the endpoint user [Fig. 3(c)]. In the context of the Canadian Optogenetics and Vectorology Foundry, new optogenetic constructs are designed by the Optogenetic Protein Engineering Core at CERVO, which are then transferred to the Viral Vector Core for assembly into a delivery vehicle that can be custom configured for the user.

**Fig. 5:**
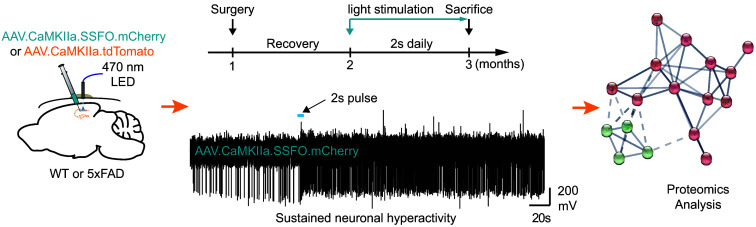
Schematics of the experimental design to generate a model of sustained hippocampal hyperactivity. Left: intrahippocampal injection of an adeno-associated virus (AAV) into the CA1 of wild-type (WT) or 5xFAD mice enables the expression of the SSFO-mCherry opsin or the fluor tdTomato (control). Center: experimental timeline (top) and an example trace recorded with a micro-optrode coupled with a 470 nm laser diode from a mouse expressing SSFO-mCherry (bottom). Right: an example interactome diagram of functional protein to protein associations generated after the proteomic analysis.

Optogenetics and opsins have emerged as a powerful method to photo-manipulate the function of individual or ensembles of neurons or glia in the central nervous system (CNS).^43^ For example, the use of optogenetics is necessary to model *in vivo* neurodegenerative disorders for which persistent neuronal activation or inhibition for several months is involved.^44^ This can be achieved using stable step-function opsin (SSFO)^45^ to generate a model of sustained neuronal hyperactivity in the rodent hippocampus (Fig. 5) that replicates several pathological aspects typical for epilepsy and Alzheimer’s disease (AD).^46^

## 4 Imaging Peripheral Nerves

**Fig. 6:**
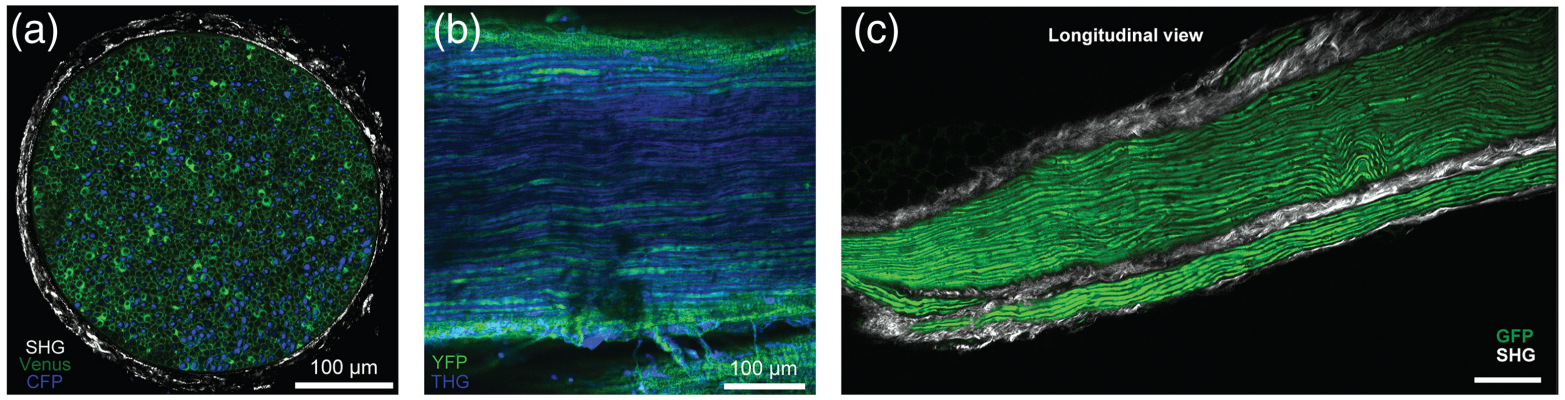
Multiphoton imaging of murine peripheral nerves. (a) A 2-photon image of in a facial nerve cross-section from Thy1-CFP/Sox10-Venus mouse. Schwann cells (Venus, green), axons (CFP, blue) and perineurial collagen (SHG, white). (b) An image of sciatic nerve from Thy1-YFP mouse 120  μm below the nerve surface showing 3-photon excitation of YFP in axons (green) and THG (blue) signals in myelin sheaths.^49^ (c) A 2-photon image of the facial nerve from ChAT-GFP mouse 50 μm below the nerve surface showing GFP in axons (green) and SHG (white) signals from the perineurium sheath. Scale bar: 50  μm.

Multiphoton microscopy enables intravital, label-free, and deep imaging of the nervous system.^47^^,^^48^ Second and third harmonic generation (SHG and THG) microscopy are powerful label-free multiphoton imaging techniques for visualizing and quantifying myelinated axons in peripheral nerve.^49^ Perineural collagen and lipid-rich structures, such as myelin sheaths, generate strong SHG and THG signals, respectively. Highly scattering components of peripheral nerves, such as collagen and myelin lipids, limit the achievable imaging depth of two-photon excitation microscopy to ∼75  μm. An ultrafast fiber laser at 1300 nm was recently used to reduce light scattering in neural tissue, resulting in doubling the penetration depth in *ex vivo* multiphoton imaging of mouse peripheral nerves (∼150  μm).^49^ Figure 6 highlights a combination of the label-free and fluorescence deep imaging capabilities of multiphoton microscopy in paraformaldehyde-fixed sectioned and whole-mounted peripheral nerves. Improvement on the imaging depth may facilitate three-dimensional histomorphometry of peripheral nerves with minimal tissue processing. Future work will further explore THG imaging at a longer illumination wavelength (1700 nm window) to increase imaging depth of nervous tissue. In addition, live and deep imaging of peripheral nerve will be obtained by combining THG with Iodixanol, an effective refractive index matching solution for *in vivo* experiments.^50^ Future development of high-peak-power lasers that produce optimal multiphoton excitation at reduced average power could help to bring THG microscopy to the clinic. In particular, *in vivo* THG could be employed on intraoperative assessment of tissue innervation and decision-making during nerve transfer surgeries.^51^

## 5 *In Vivo* Imaging of Rodent Cerebral Cortex

### 5.1 Super-Resolution Imaging *in Vivo*

Super-resolution imaging methods have shattered the diffraction limit of optical microscopy. Among them, stimulated emission depletion (STED) microscopy is a deterministic super-resolution modality based on point-scanning fluorescence imaging and spatial beam shaping.^52^ STED has been successfully used in neuroscience for more than fifteen years, offering diffraction-unlimited visualization of protein distributions in fixed tissues but also volumetric live imaging of neuronal morphology in various animal models and preparations.^53^^,^^54^ Yet, its application to the brain of a living animal remains technically challenging, to the extent that the perfect STED image generally remains a distant and elusive target.

**Fig. 7:**
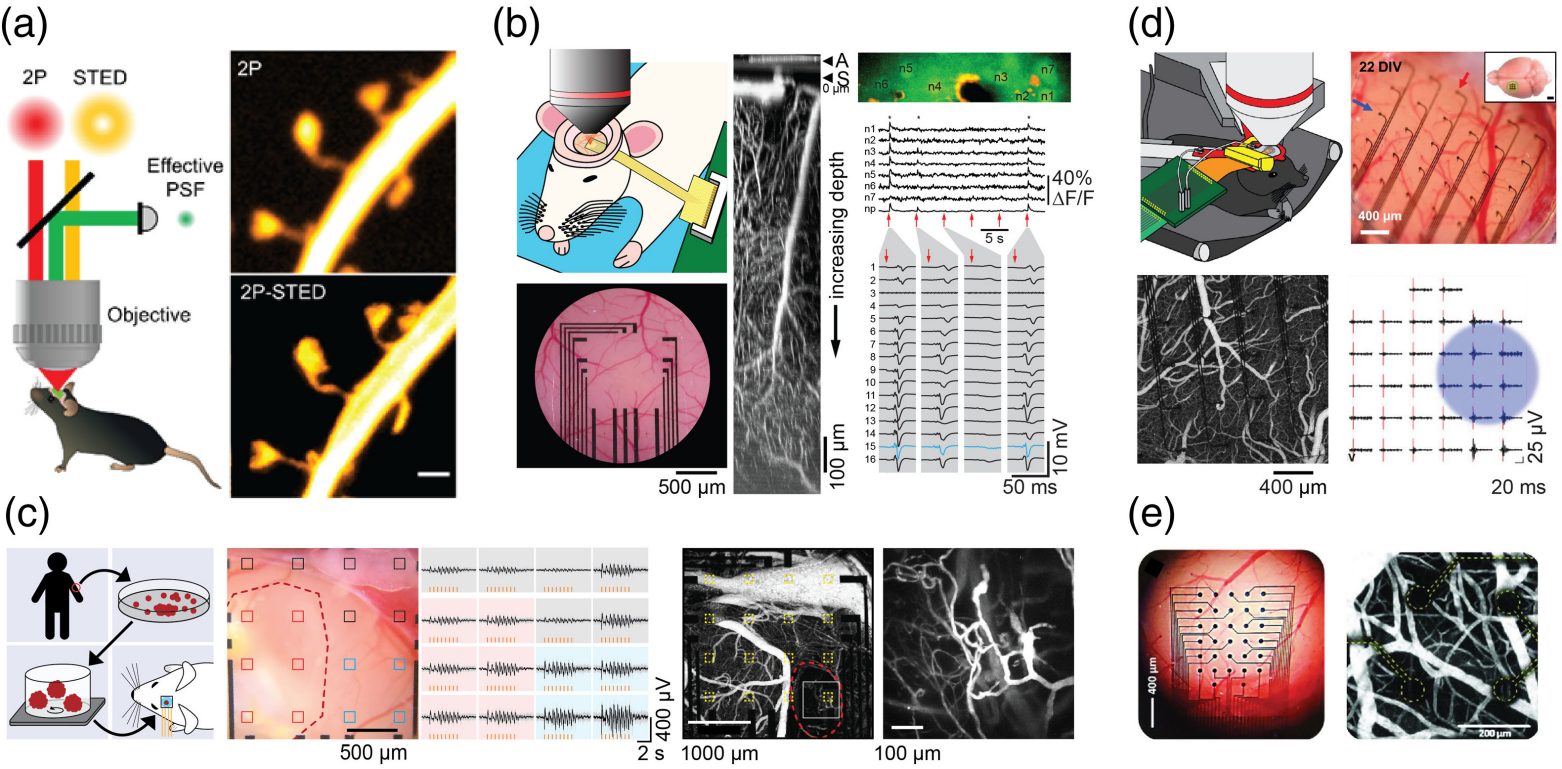
*In vivo* imaging of the mouse cerebral cortex. (a) Schematic of the *in vivo* 2P-STED microscopy configuration. Regular 2-photon and 2P-STED image of a segment of dendrite in the hippocampus of a Thy1-GFP mouse illustrating the gain in spatial resolution. Scale bar: 1  μm. (b) Two-photon imaging of cortical microvasculature and neuronal calcium activity through transparent optically graphene microelectrode arrays (gMEAs). Left: schematic of the setup (top) and bright field image of surface gMEA (bottom). Electrodes in the center are invisible, gold wires are seen in black. Middle: XZ projection of Alexa 680-labeled vasculature below an electrode (A) from the cortical surface (S) to a depth of ∼800  μm. *Right*: LFP in response to stimulation of the whisker pad (red arrows) acquired simultaneously with imaging (top) of OGB1-loaded neurons (n1-n7) underneath electrode 15 (blue traces). Adapted from Ref. 59. (c) Two-photon imaging and gMEAs for investigation of human iPSC-derived cortical organoids implanted into mouse cortex. Left: experimental approach. Center: LFP recorded above mouse cortex (blue) and organoid (red) in response to a visual stimulus. Right: low- and high-magnification vascular images; organoid borders are outlines in red. Adapted from Ref. 60. (d) A silicon microneedle electrode array (SiMNA) with transparent backing. Top left: experimental configuration. Top right: bright field image of the array. Bottom left: two-photon image of the surface vasculature. Bottom right: multi-unit activity elicited by optogenetic stimulation of excitatory neurons. The blue spot shows stimulated area. Adapted from Ref. 61. (e) Platinum nanorod (PtNR) surface microelectrode arrays with transparent backing. Left: bright field image of the array. Right: two-photon image of the surface vasculature. Adapted from Ref. 62.

To date only a handful of studies have ventured into the *in vivo* realm, establishing proof-of-concept of longitudinal monitoring of nanoscale structures in the cortex^55^^–^^57^ and hippocampus^58^ of anesthetized mice [Fig. 7(a)]. For *in vivo* super-resolution imaging, the sample is viewed through a cranial window,^63^ where a piece of the skull is cut away and replaced with a glass coverslip, to provide optical access to the brain situated right underneath. This imposes the use of relatively long working distance objectives, at the expense of the numerical aperture. Additionally, a certain amount of imaging depth is required to reach the structures of interest, and blood pulsations and breathing tend to blur the images.^57^ Lastly, STED performance crucially depends on the shape and quality of the point-spread function (PSF) of the depletion beam, which is very sensitive to optical aberrations of the STED light passing from the microscope to the brain via the cranial window. The resulting mismatch in refractive index, along with potential imperfect optical alignment, distort the wavefront and subsequently reduce spatial resolution and signal-to-noise ratio.^64^^–^^66^

The geometry and positioning of the cranial window under the microscope are very important. For instance, a tilt of the coverslip relative to the optical axis by just 1° can dramatically distort the PSF.^67^ Ongoing optimization of the surgical procedures is bound to significantly improve image quality and stability. In parallel, the penetration depth of STED microscopy was increased from just a few to tens of microns by means of 2-photon excitation and the use of new objective lenses with high numerical apertures, long working distances, and correction collars.^68^^,^^69^ The use of adaptive optics to pre-compensate PSF distortions makes it possible to further increase the depth penetration of super-resolution imaging in live conditions.^57^^,^^64^^,^^66^ Motion-induced artifacts are a common problem for *in vivo* imaging, in particular for STED. While *post hoc* correction offers some remedy, it may require active motion correction strategies^70^ to reduce motion blur sufficiently. Wide-field illumination based on optical lattices^71^^,^^72^ has emerged as a promising strategy to increase temporal resolution and throughput. Lastly, the relatively high laser power required for STED microscopy makes it prone to photo-bleaching and phototoxicity.^73^ Thus, STED microscopy can certainly benefit from the development of brighter and more photostable fluorescent probes. Various technically sophisticated solutions have been developed to mitigate this issue (*e.g.* RESCue, DyMin, MinField),^74^ but they remain to be demonstrated *in vivo*. More recently, an alternative and rather straightforward approach sidesteps the bleaching and toxicity issue almost entirely, namely super-resolution shadow imaging (SUSHI).^75^ It is based on 3D-STED microscopy and inverse labelling of the brain tissue, where a diffusible, yet membrane-impermeable, fluorescent dye is added to the interstitial fluid. SUSHI generates high-contrast and bleaching-insensitive “negative imprints” of all cellular structures, while at the same time also visualizing the extracellular space (ECS) of the brain, which is of considerable interest for neurobiologists.

Leveraging various strands of technical developments, the prospects for STED are very bright, widening and solidifying its scope and performance for nanoscale investigations of complex biological systems.

### 5.2 *In Vivo* Imaging Through Microelectrode Arrays

In recent years, diverse strategies have been developed to merge *in vivo* neurophotonics with *in vivo* neuroelectronics, including transparent surface electrode arrays and penetrating electrode shanks with integrated light sources, light guides, and photodetectors.^76^^–^^84^ For optical modalities that interface with brain tissue through large numerical aperture objectives that have a wide footprint and working distance of a few millimeters, unobstructed access to the surface is necessary. One such modality is 2-photon microscopy. Therefore, combination of 2-photon imaging with neurorecording devices requires that electronic circuit boards are positioned on the side and not immediately above the electrodes as customary for extracellular neurophysiology. Figures 7(b)–7(c) illustrate 2-photon imaging though transparent graphene microelectrode arrays (gMEA) placed on the cortical surface.^59^^,^^85^ The presence of the array does not impede the penetration depth and minimally degrades resolution of 2-photon imaging. Likewise, the infrared laser used for 2-photon imaging and blue laser used for optogenetic stimulation cause minimal photovoltaic artifacts in electrophysiological recordings, as long as the imaging laser is not focused on the electrode pad [Fig. 7(b)]. In another study, the same multimodal setup was used to investigate integration of human cortical organoids, derived from induced pluripotent stem cells (iPSCs), into the mouse cortex^60^ [Fig. 7(c)]. Stimulation of the mouse with white-light flashes to the contralateral eye evoked local field potentials (LFP) and spikes (multiunit activity, MUA) in both the mouse cortex and the implanted organoid, suggesting the development of functional connections between the host (mouse) and xenograft (human) neurons. In the same study, 2-photon vascular imaging through gMEA was used to confirm vascularization of the engrafted organoid by the host blood vessels [Fig. 7(c)].

Another strategy to engineer electrode arrays compatible with 2-photon imaging is to use nontransparent microelectrodes that are small enough, such that their shadows virtually disappear with penetrating depth.^86^ Figure 7(d) illustrates one such case where an array of poly(3,4-ethylenedioxythiophene) poly(styrene sulfonate) (PEDOT:PSS)-coated silicone microneedles was manufactured on a flexible and transparent substrate to allow simultaneous 2-photon imaging and optogenetic stimulation.^61^ Another example is platinum nanorod electrodes on a transparent substrate^62^ [Fig. 7(e)]. The electrochemical properties of these non-transparent materials allow reducing the electrode size to single microns while maintaining the low impedance necessary to sense not only LFP but also spikes.

As for biosensors for calcium and voltage, the choice of a neurorecording device depends on the application. The weighting considerations include dimensionality (e. g., surface electrode grids or linear penetrating arrays), measurement resolution (pitch), sensitivity (electrode size and material properties), spatial coverage, stability in a chronic setting and geometrical constraints of specific optical modalities.

## 6 Conclusion

In recent years, advances in neurophotonic technologies^82^ have been instrumental in gaining deeper understanding of how neurons and neural circuits work and govern our behavior.^87^^,^^88^ In the next decade, we expect that further developments in synthetic chemistry, protein engineering, and parallel advances in optical instrumentation will push our ability to detect, measure, manipulate, and follow the intricate components of the central and peripheral nervous system. This basic knowledge will increase our ability to design novel treatments for neurological and psychiatric disorders. Along the way, invaluable opportunities for sharing, training, and cross-pollination will be awaiting at summits and summer schools such as Frontiers in Neurophotonics. We look forward to seeing you there!

## Data Availability

Data sharing is not applicable to this article, as no new data were created or analyzed.
